# Cover cropping enhances fruit quality in protected citrus cultivation by modulating rhizosphere microbiome and iron availability

**DOI:** 10.3389/fpls.2026.1836783

**Published:** 2026-06-18

**Authors:** Lile Deng, Ying’an Pan, Hongfei Li, Liming Wu, Ce Wang, Dongxin Liu, Junli Zhang, Jiajun Cheng, Fang Song, Zhiyong Pan

**Affiliations:** 1 National Key Laboratory for Germplasm Innovation & Utilization of Horticultural Crops, College of Horticulture and Forestry Sciences of Huazhong Agricultural University, Wuhan, China; 2 Hubei Key Laboratory of Germplasm Innovation and Utilization of Fruit Trees, Institute of Fruit and Tea, Hubei Academy of Agricultural Science, Wuhan, China

**Keywords:** citrus, cover cropping, fruit quality, iron availability, rhizosphere microbiome

## Abstract

**Introduction:**

Citrus is one of the most widely cultivated fruit trees worldwide, and protected cultivation has become increasingly prevalent in recent years. Cover cropping improves orchard soil health, yet its mechanisms in protected citrus cultivation remain unclear. This study investigated how white clover (*Trifolium repens* L.) and ryegrass (*Lolium perenne* L.) affect soil properties, rhizosphere microbiota, and fruit quality in greenhouse-grown ‘Kanpei’ citrus through integrated analyses of soil physicochemical properties, high-throughput amplicon sequencing, and microbial isolation.

**Results:**

Both cover crops significantly increased total soluble solids (TSS) and vitamin C levels in mature fruits. Ryegrass enhanced the availability of nitrogen, phosphorus, calcium, magnesium, and manganese, whereas white clover more effectively acidified the soil and increased iron (Fe) availability. Each cover crop distinctively altered the rhizosphere microbial community. Notably, white clover specifically enriched Pseudomonas, which strongly correlated with elevated soil available Fe, TSS, and vitamin C. Screening with Chrome Azurol S (CAS) agar identified Pseudomonas as the dominant siderophore-producing genus. Inoculation with a representative strain, *Pseudomonas* sp. PA9, significantly enhanced Fe uptake, chlorophyll content, and fruit quality, offering insights into its potential role in promoting fruit quality under protected cultivation.

**Conclusions:**

This work provides a comprehensive understanding of how white clover promotes fruit quality via fostering siderophore-producing Pseudomonas that enhance Fe mobilization, suggesting new avenues for developing microbiome-based management strategies in protected citrus cultivation. These findings underscore the potential of cover crop-mediated microbial recruitment in advancing sustainable citrus production and soil health improvement.

## 1 Introduction

Protected cultivation, employing structures such as greenhouses and polytunnels, offers a powerful means to regulate growing environments and facilitate citrus production in non-traditional regions or seasons (Dong et al., 2024; Graham et al., 2024). However, these systems also face intrinsic sustainability challenges. Intensive management practices, including clean tillage, monocropping, and excessive application of chemical fertilizers and pesticides, can degrade soil health, leading to compaction, reduced organic matter, and diminished microbial diversity (Cerdà et al., 2022). Such degradation constrains nutrient availability and root function, ultimately impairing orchard productivity and fruit quality (Liu et al., 2024; Zheng et al., 2024).

Harnessing beneficial rhizosphere microorganisms represents a promising strategy to reverse this trend. A healthy rhizosphere microbiome can enhance nutrient acquisition (Qiao et al., 2024; Ren et al., 2025), stress tolerance, and fruit quality (Fu et al., 2025; Zhao et al., 2024) by modulating root function and soil properties (Li et al., 2026; Zhong et al., 2022). Thus, identifying management practices that foster beneficial microbial communities is critical for advancing sustainable citrus production. Orchard cover cropping, the cultivation of inter-row herbaceous plants, has gained attention for its ability to improve soil nutrient cycling (Duan et al., 2024; Mia et al., 2020), revitalize microbial communities (Chinthalapudi et al., 2026; Guo et al., 2024), and enhance fruit quality (Jiao et al., 2024; Lidoy et al., 2025). Evidence suggests that cover plants can reshape the rhizosphere environment, promoting the enrichment of beneficial bacteria that support tree nutrition and fruit attributes (Abobatta, 2020; Fang et al., 2022). Nevertheless, how cover cropping influences rhizosphere microbiota and fruit quality under protected citrus cultivation, where environmental control and soil use are distinct, remains poorly understood.

In this study, we evaluated the effects of two cover crops, white clover (*Trifolium repens* L.) and ryegrass (*Lolium perenne* L.), compared to clean tillage, on fruit quality, soil properties, and the rhizosphere bacterial community in greenhouse-grown ‘Kanpei’ citrus. We combined high-throughput sequencing with chemical soil analysis to identify cover crop-induced shifts in microbial structure and nutrient availability. Bacterial strains showing strong correlations with soil available iron (Fe) and fruit quality parameters were isolated using Chrome Azurol S (CAS) screening. Finally, we validated the role of a representative siderophore-producing *Pseudomonas* strain PA9 in promoting Fe uptake and fruit quality via rhizosphere inoculation.

## 2 Materials and methods

### 2.1 Experimental treatments and fruit quality measurement

The experiment was conducted at a citrus orchard in Huaijiagou Village, Junxian Town, Danjiangkou City, Shiyan City, Hubei Province, China (32°40′27.18″N, 111°31′1.66″E). Eight-year-old ‘Kanpei’ citrus trees, exhibiting uniform growth vigor and no signs of disease or pest infestation, were used as plant material. The trees were grown in a multi-span greenhouse with a steel frame and plastic film cover. At the initiation of the experiment, three treatments were established within the greenhouse: white clover (*Trifolium repens* L.) cover cropping, ryegrass (*Lolium perenne* L.) cover cropping, and clean tillage (control). For each treatment, three ‘Kanpei’ citrus trees with good health and consistent vigor were selected, with each tree serving as one biological replicate (n=3 per treatment). Cover crop seeds were broadcast in April 2023. Citrus rhizosphere soil samples were collected in June, September, and November 2023, and January 2024 for subsequent analysis of microbial community composition and physicochemical properties. Citrus fruits were harvested at November 2023 (color-turning stage) and January 2024 (maturity stage) for fruit quality assessment.

Individual fruit weight was measured using an electronic analytical balance (accuracy 0.01 g). Fruit total soluble solids (TSS) content was determined using a PAL-1 portable digital refractometer. Titratable acidity (TA) and vitamin C (Vc) content were measured by indicator titration methods (Wu et al., 2017).

### 2.2 Rhizosphere soil sampling and physicochemical analysis

Random sampling was conducted at the drip line of citrus plants. Weeds and surface soil at the sampling points were removed using a shovel, and samples were collected from the depth interval of 10–30 cm, which was rich in roots approximately 1 mm in diameter. The soil blocks containing abundant fine roots were placed into sterile ziplock bags, kept on ice, and promptly transported back to the laboratory for processing. roots with rhizosphere soil removed were transferred into 50 ml centrifuge tubes containing pre-chilled PBS, and root-attached soil was collected using ultrasonic vibration. The ultrasonic cleaner was filled with pre-cooled (4 °C) deionized water, and the centrifuge tubes were immersed in the water for 40 s of ultrasonic treatment (Bai et al., 2015). The fine roots were then taken out from the tubes with sterile tweezers, air-dried in a laminar flow hood, and stored at -80 °C for subsequent experiments. The centrifuge tubes containing only the soil suspension were centrifuged at 12,000 × g for 1 min at 4 °C. After discarding the supernatant, one portion of the pellet was stored at -80 °C for microbiome analysis, while the other portion was air-dried in a fume hood for one week. After drying, the soil clumps were gently crushed with a wooden stick and passed through a 20-mesh sieve.

For mineral element analysis, approximately 2 g of rhizosphere soil was extracted using MIII solution. The concentrations of phosphorus (P), potassium (K), calcium (Ca), magnesium (Mg), iron (Fe), and manganese (Mn) were determined using an Inductively Coupled Plasma Optical Emission Spectrometer (ICP-OES, Finnigan MAT, Element I, Germany). Nitrogen (N) content was analyzed using an elemental analyzer (Elementar Vario MACRO, Germany) (Huang et al., 2024). Iron content in citrus leaves and roots was measured using a commercial tissue iron assay kit (Zhou et al., 2024).

### 2.3 Analysis of rhizosphere soil microbial community

Rhizosphere soil samples were processed for 16S rRNA gene amplicon sequencing targeting the V3-V4 hypervariable regions using primer pair 341F/806R on an Illumina NovaSeq platform at Personalbio Biotechnology Co., Ltd. (Shanghai, China) (Niu et al., 2017). Raw sequencing data were processed on the Personalbio GeneCloud platform using QIIME2 (version 2019.4). Briefly, primer sequences were removed with qiime cutadapt trim-paired and unprimed reads discarded. Denoising, quality filtering, read merging, and chimera removal were performed using the DADA2 pipeline (qiime dada2 denoise-paired), applied separately to each sequencing library. After processing all libraries, ASV (amplicon sequence variant) feature tables and representative sequences were merged across libraries. Singletons (ASVs with a total abundance of 1 across all samples) were removed. The final ASVs ranged from approximately 410 to 430 bp, consistent with the expected length of the V3-V4 amplicon after primer trimming. Taxonomic assignment was performed against the Silva database (version 132). For downstream analyses, the ASV table was rarefied to an even depth of 30,000 reads per sample (the minimum sequence count after filtering). Based on the normalized ASV abundance table, Bray-Curtis dissimilarities were calculated and non-metric multidimensional scaling (NMDS) was performed using the vegan package in R to visualize differences in microbial community structure among samples. The goodness of fit of the ordination was assessed using the stress value. Microbial α-diversity was evaluated with the Chao1 index (estimating species richness with bias correction for undetected taxa) and the Shannon index (integrating richness and evenness). All indices were derived from normalized abundance tables using appropriate R packages.

### 2.4 Microbial isolation and identification

All experimental procedures were conducted under aseptic conditions within a laminar-flow hood. For each treatment, 1 g of composite rhizosphere soil was suspended in 50 mL of sterile distilled water in a sterile 50 mL centrifuge tube and vortexed thoroughly. A 10 μL aliquot of the resulting turbid suspension was spread evenly onto Chrome Azurol S (CAS) agar plates (Liu et al., 2018). The plates were sealed with parafilm and incubated at 28 °C in the dark for 48 h. Following incubation, distinct colonies were picked and inoculated into 1.5 mL microcentrifuge tubes containing 600 μL of LB broth. Cultures were incubated at 28 °C with shaking at 200 rpm for 48 h until reaching robust growth. For bacterial identification, the nearly full-length 16S rRNA gene was amplified using universal bacterial primers 27F and 1492R. PCR products were confirmed by agarose gel electrophoresis, and amplicons of the expected size were submitted to Personalbio Biotechnology Co., Ltd. (Shanghai, China) for Sanger sequencing and subsequent taxonomic classification.

### 2.5 Functional validation of bacterial strain

Ten-month-old trifoliate orange (Poncirus trifoliata) seedlings were planted in soil from the clean tillage treatment in a plastic greenhouse. Eight seedlings constituted one replicate (n = 3 replicates per group). Seedlings were inoculated weekly with 5 mL per plant of PA9 bacterial suspension (OD_600_ = 0.2). After four weeks, leaf SPAD value, soil available iron, and leaf iron content were measured. For field validation, ‘Kanpei’ citrus trees at fruit color-turning stage were selected, with three trees per group (n = 3). After initial leaf sampling, PA9 suspension (OD_600_ = 0.2, diluted from an OD_600_ = 2 laboratory stock with sterile water) was applied at 10 L per tree to the absorbing root zone (30–50 cm from the trunk) via shallow trenches along the drip line or radial furrows, avoiding root damage. After slow and even application, trenches were rinsed with clean water, backfilled, lightly compacted, and mulched. At fruit maturity, fruits were harvested for quality assessment, and leaves and roots were collected for chlorophyll and iron analysis.

### 2.6 Data analysis

Graphs for fruit quality, soil physicochemical properties, and leaf/root mineral element data were generated using GraphPad Prism software. One-way analysis of variance (ANOVA) was performed using SPSS 25.0 software, followed by the Least Significant Difference (LSD) test for multiple comparisons. The significance level was set at *P < 0.05. Data from the rhizosphere soil microbial community analysis were downloaded from the Personalbio GeneCloud platform, and relevant graphs were generated using the online tools provided.

## 3 Results

### 3.1 Cover cropping enhances citrus fruit quality

**Figure 1:**
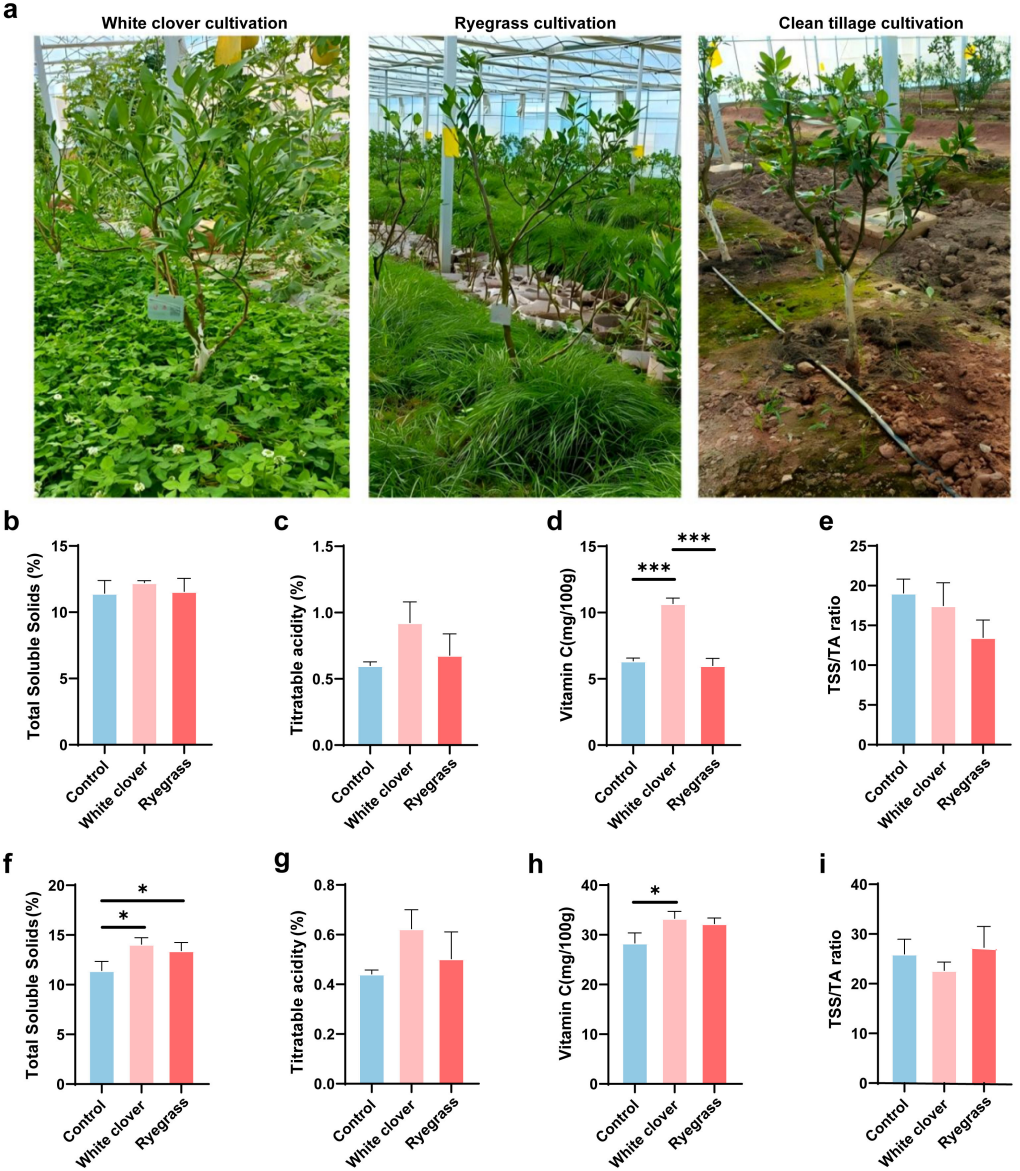
Effects of different cover cropping management practices and periods on citrus fruit quality. **(a)** Field view of the cover cropping system in a greenhouse. **(b–e)** Fruit quality during the fruit color-turning period (November 2023) of citrus under polytunnel cover cropping (white clover, ryegrass) and clean tillage. **(f–i)** Fruit quality during the fruit ripening period (January 2024) of citrus under polytunnel cover cropping and clean tillage. Data are presented as mean ± standard error (SE). Statistical significance was determined by one-way ANOVA followed by Tukey’s HSD post-hoc test. *P<0.05; ***P<0.001. Different lowercase letters indicate significant differences among treatments at the same stage (P<0.05).

To compare the effects of different cover crops on the orchard floor, photographs of the three treatments were taken during the experiment (**Figure 1a**). White clover and ryegrass were established as living mulches, while clean tillage served as the control. At the fruit color-turning stage (November 2023), no significant difference was observed in total soluble solids (TSS) among treatments (**Figure 1b**). Compared with clean tillage, white clover cover led to a non-significant increase in titratable acidity (TA), while ryegrass cover showed no significant difference (**Figure 1c**). Under white clover cover, vitamin C (Vc) content was significantly higher than under clean tillage, and the TSS/TA ratio showed a non-significant increasing trend. Under ryegrass cover, Vc content did not change significantly, whereas the TSS/TA ratio showed a non-significant decreasing trend and was significantly lower than that under white clover cover (**Figures 1d, e**). By the maturity stage (January 2024), TSS levels were highest under white clover and second highest under ryegrass, both being significantly higher than under clean tillage (**Figure 1f**). TA remained significantly highest under white clover (**Figure 1g**). Regarding Vc content, only white clover cover was significantly higher than clean tillage, while ryegrass cover did not differ significantly from clean tillage (**Figure 1h**). No significant differences in TSS/TA ratio were detected among treatments at maturity (**Figure 1i**). Overall, cover cropping significantly increased TSS content in mature fruits, and white clover also significantly increased Vc content.

### 3.2 Cover cropping alters soil properties and microbial composition

**Figure 2:**
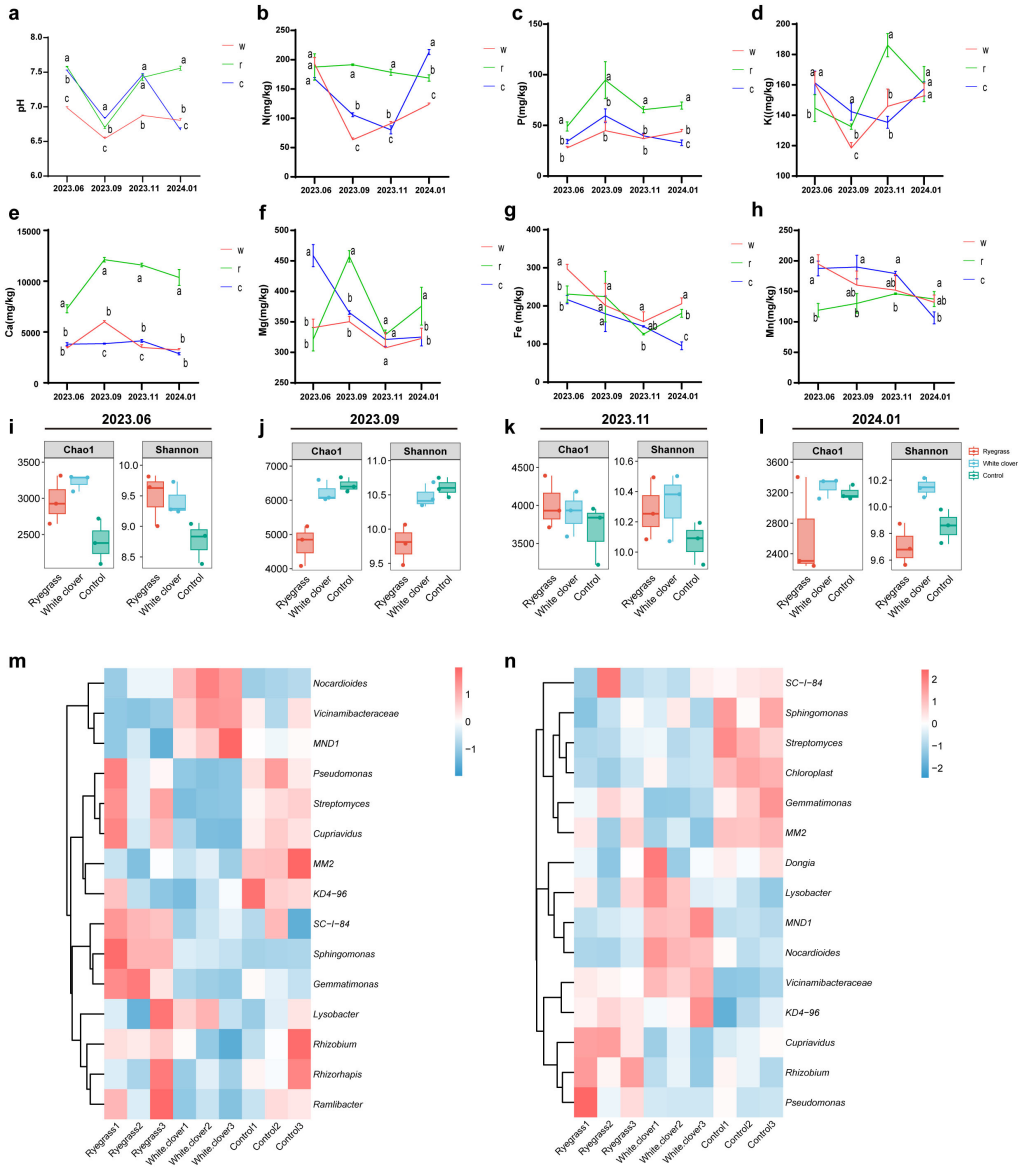
Effects of different cover cropping management practices and sampling periods on soil physicochemical properties and rhizosphere microorganisms. **(a–h)** Temporal variations in soil physicochemical properties [pH, nitrogen (N), phosphorus (P), potassium (K), calcium (Ca), magnesium (Mg), iron (Fe), and manganese (Mn)] under three treatments: white clover cover cropping, ryegrass cover cropping, and clean tillage. Different lowercase letters **(a–c)** denote significant differences among treatments at each sampling time point, determined by one-way analysis of variance (ANOVA) followed by Tukey’s post-hoc multiple comparison test (P < 0.05). Error bars represent the standard error of the mean (SEM). **(i–l)** Box plots of Chao1 index (species richness) and Shannon index (species diversity) of citrus rhizosphere soil microbial communities across sampling periods under different treatments. For box plots, the middle horizontal line represents the median; the box denotes the 25th-75th percentile range; whiskers indicate the minimum and maximum non-outlier values; scattered dots represent outliers. P-values shown in the panels represent overall differences among three treatments determined by one-way ANOVA (no post-hoc pairwise comparisons were performed). **(m, n)** Heatmaps showing differences in rhizosphere soil bacterial genus abundance between cover-cropped and clean-tilled citrus under greenhouse conditions during the fruit coloring period **(m)** and fruit maturation period **(n)**. Bacterial genus abundance data were normalized using the Z-score standardization method for heatmap visualization.

Throughout the experiment, rhizosphere soil pH under white clover remained below 7 (**Figure 2a**). Ryegrass significantly increased alkali-hydrolyzable nitrogen (September and November 2023), available calcium (whole period), available phosphorus (whole period), and available magnesium (September 2023 and January 2024). It also elevated available manganese at maturity. These results indicate ryegrass enhanced the availability of N, P, Ca, Mg, and Mn, while white clover was more effective in acidifying soil and improving Fe availability (**Figure 2b–h**).Available iron (Fe) was significantly higher under white clover in June 2023 and January 2024 than under clean tillage. From color-turning to maturity, available P and Fe under cover crops increased consistently, becoming significantly higher than under clean tillage by harvest (**Figure 2c, g**).

Both cover crops increased microbial richness and diversity compared to clean tillage in June 2023 and January 2024, with differences most pronounced in June (**Figure 2i–l**). Microbial community composition also differed significantly among treatments (**Supplementary Figure S1**). At color-turning, ryegrass enriched *SC-I-84*, *Sphingomonas* and *Gemmatimonas*, whereas white clover enriched *Nocardioides*, *Vicinamibacteraceae* and *MND1* (**Figure 2m**). At maturity, ryegrass-associated communities shifted to *Cupriavidus*, *Rhizobium* and *Pesudomonas*, whereas white clover communities remained dominated by *MND1*, *Nocardioides* and *Vicinamibacteraceae* (**Figure 2n**). These findings confirm that cover cropping reshapes the rhizosphere microbiota in a stage-dependent manner.

### 3.3 Correlations among fruit quality, soil properties, and microbiota

From color-turning to maturity, fruit TSS correlated positively with soil available P and Fe. TA correlated negatively with alkali-hydrolyzable N and positively with available Mn. Vc correlated positively with available Fe and negatively with available Mn. Both TSS and Vc showed significant positive correlations with available Fe **Supplementary Figure S2**), indicating a potential role of Fe in fruit quality. These findings are consistent with previous studies showing that the rhizosphere microbiome promotes fruit sugar accumulation by improving iron availability through siderophore-producing bacteria (Wu et al., 2026).

**Figure 3:**
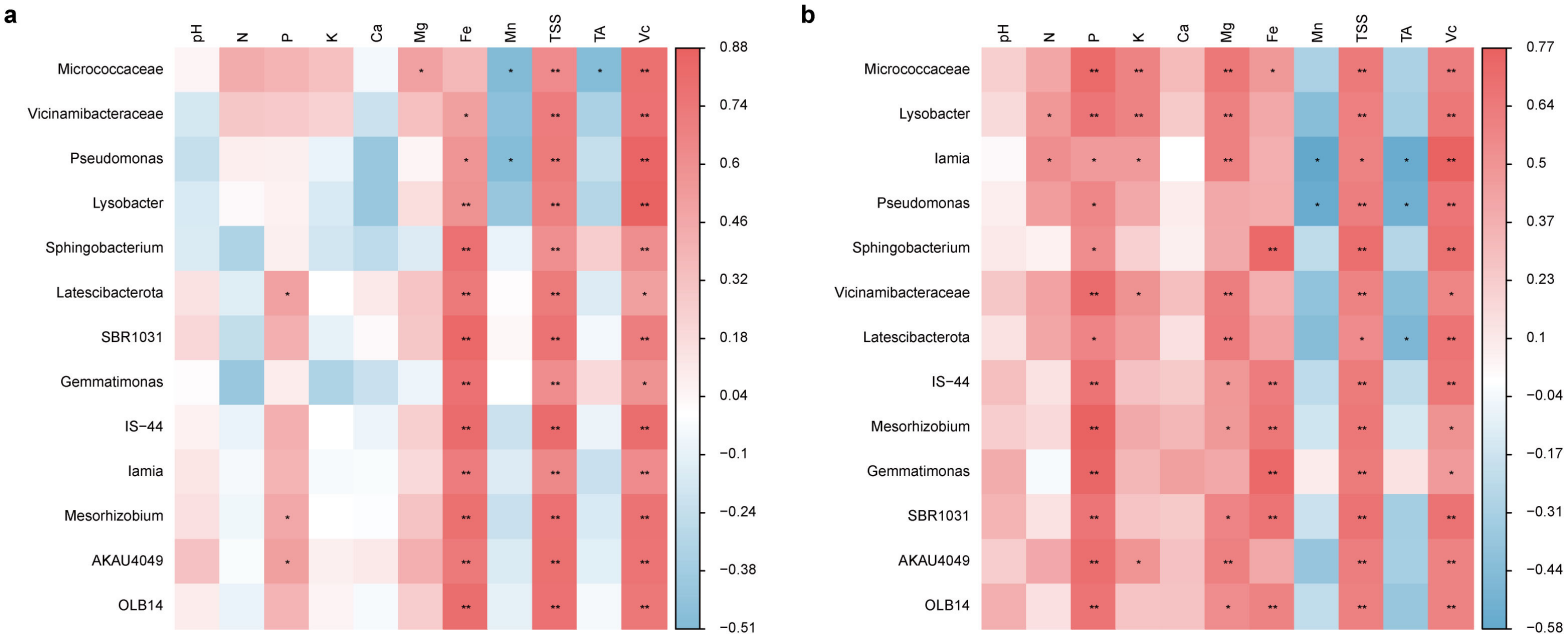
Correlation analysis of rhizosphere soil microorganisms with fruit quality in Citrus. **(a)** Under white clover cultivation treatment, 13 microbial species were significantly correlated with fruit quality. **(b)** Under ryegrass cultivation treatment, 13 microbial species were significantly correlated with fruit quality. * and ** indicate significant correlations at the 0.05 and 0.01 levels, respectively.

MetagenomeSeq analysis (ASV frequency≥0.3) identified cover crop-enriched bacterial genera. Under white clover, TSS correlated positively with 74 genera (e.g., KF-JG30-B3, *Pseudomonas*), TA positively with 7 genera (e.g., *Rhodococcus*) and negatively with unclassified_*Micrococcaceae*, and Vc positively with 66 genera (e.g., Dongia, *Pseudomonas*) and negatively with *Sphingomonas* **Supplementary Table 1**). Under ryegrass, TSS correlated positively with 37 genera (e.g., *Pseudomonas*, *Sphingobacterium*), TA positively with *Novosphingobium* and negatively with *Pseudomonas*, and Vc positively with 22 genera (e.g., *Pseudomonas*) and negatively with others including *Novosphingobium* **Supplementary Table 2**). Thirteen genera (e.g., *AKAU4049*, *Pseudomonas*, *Lysobacter*) were consistently positively correlated with TSS and Vc under both cover crops (**Figures 3a, b**).

Correlations between enriched genera and soil properties also differed by treatment. Under white clover, available Fe correlated positively with 80 genera (e.g., *Pseudomonas*, *Luteimonas*), and available P with 16 genera (e.g., *Rhodococcus*) **Supplementary Table 3**). Under ryegrass, available P correlated positively with 97 genera (e.g., *C0119*, *Luteitalea*), available K with 96 genera (e.g., *Amycolatopsis*, *Ellin6055*), available Ca with 73 genera (e.g., *SWB02*, *Pseudonocardia*), and pH with 29 genera (e.g., *TM7a*, *RB41*) (**Supplementary Table 4**).

Thirteen bacterial genera were co-enriched under both cover crops (e.g., *AKAU4049*, *Lysobacter*, *Pseudomonas*, *Gemmatimonas*) and positively correlated with TSS and Vc (**Figures 3a, b**). Under white clover, these genera correlated mainly with available Fe (**Figure 3a**); under ryegrass, they correlated with available P, K, and Ca. Four genera (*Lysobacter*, *Pseudomonas*, *Vicinamibacteraceae*, and *Gemmatimonas*) were consistently abundant across growth stages and positively correlated with soil available Fe and fruit quality (**Figure 3b**).

### 3.4 Screening and validation of fruit quality-associated microbes

Using CAS medium, 154 strains were isolated from the rhizosphere, with 89 successfully sequenced. *Pseudomonas* dominated (79.78%), followed by *Burkholderia* (7.87%), *Achromobacter* (4.49%), and *Priestia* (2.25%); other genera each constituted <1.5% **Supplementary Figure S3a**). Among all genera showing positive correlations with soil available Fe, TSS, and Vc content, only *Pseudomonas* was identified within this culturable fraction.

Nine *Pseudomonas* species were identified (PA1-PA9), with PA2 (40.85%) and PA9 (38.03%) being most abundant **Supplementary Figure S3b**). Abundance of PA9 correlated positively with soil available Fe, TSS, and Vc **Supplementary Figure S3c**). Five ASVs enriched under cover crops showed ≥97% similarity to PA9 and were classified as such **Supplementary Table 5**). The PA9 abundance was significantly higher under cover crops than clean tillage, highest under white clover **Supplementary Figure S3d**).

**Figure 4:**
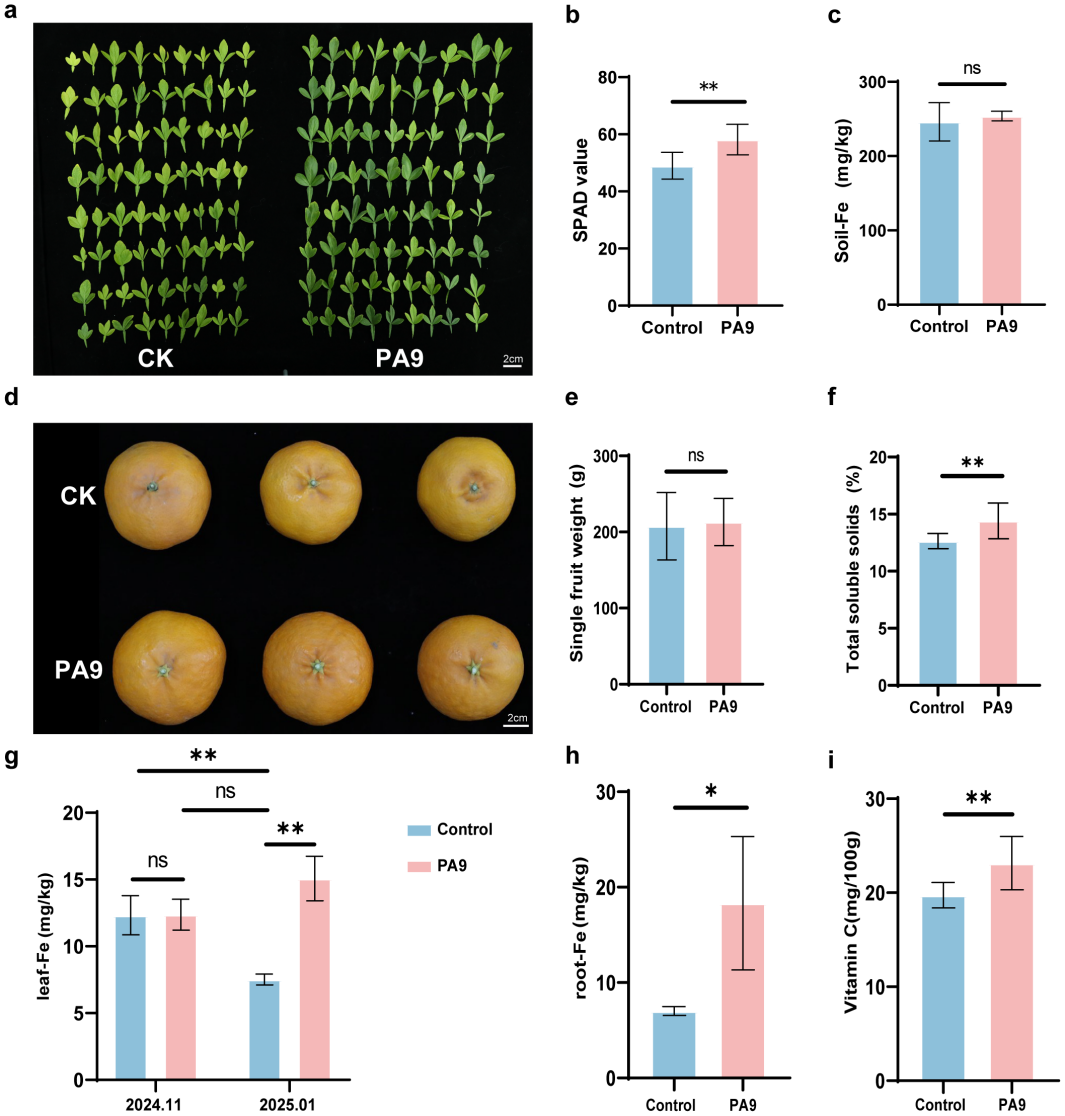
Functional verification of *Pseudomonas* PA9. **(a–c)** Leaf and rhizosphere soil status in citrus seedlings treated with PA9 strain. **(d)** Phenotype of citrus fruits after PA9 treatment. **(e)** Changes in single fruit weight of citrus fruits after PA9 treatment. **(f)** PA9 treatment significantly increased the soluble solids content of citrus fruits. **(g)** Effects of PA9 bacterial treatment on leaf iron content of citrus at different stages. **(h)** Effects of PA9 bacterial treatment on root iron content of citrus at ripening stage. **(i)** PA9 treatment significantly increases the Vc content of citrus fruits. *P<0.05; **P < 0.01 (Student’s *t*-test). ns indicates no significant difference (p > 0.05).

Inoculation of *Poncirus trifoliata* seedlings with PA9 significantly increased leaf chlorophyll content (SPAD) and showed an upward trend in soil available Fe (**Figures 4a–c**). In field trials, PA9 treatment significantly enhanced fruit TSS and Vc, as well as root iron (Fe) content, although it had no significant impact on individual fruit weight (**Figures 4d–i**). Although leaf Fe concentration did not differ between treated and control citrus trees at the color-turning stage, it became significantly higher in treated plants at maturity. Notably, leaf Fe decreased from color-turning to maturity in control plants, whereas it increased in PA9-treated plants. These results are consistent with the possibility that PA9 influences iron uptake, leaf iron retention, and fruit quality, although causal mechanisms remain to be established.

## 4 Discussion

White clover and ryegrass cover crops exerted contrasting effects on citrus rhizosphere soil properties, indicative of distinct nutrient activation strategies. In particular, the continuous acidification of soil (pH < 7) under white clover cultivation may promote iron (Fe) mobilization by increasing its solubility. This likely contributed to the significantly higher available Fe levels observed under white clover, particularly in June 2023 and January 2024. In contrast, ryegrass enhanced the availability of multiple macro- and micronutrients (N, P, Ca, Mg, Mn), likely through root-mediated weathering and carboxylate exudation (Liu et al., 2011). The sustained increase in available P under both cover crops from fruit color-turning to maturity (**Figures 2a–h**) underscores their role in promoting nutrient retention and supply during critical phenological stages, contrasting with the nutrient decline under clean tillage.

Both cover crops significantly increased rhizosphere microbial diversity, with the most pronounced effects observed in June 2023. This aligns with established evidence that cover crops provide diverse carbon sources and ecological niches, fostering richer microbial communities than disturbance-prone clean tillage systems (Molnár et al., 2023). More importantly, white clover and ryegrass drove distinct, stage-specific shifts in microbial community composition. White clover enriched bacterial genera such as *Streptomyces* and *Sphingomonas* at the color-turning stage, followed by a marked enrichment of *Pseudomonas* and *Cupriavidus* at maturity. This successional pattern suggests a dynamic interaction between white clover’s rhizosphere processes (e.g., acidification, exudation) and microbial recruitment. Recent studies further suggest that host plant metabolic traits and genotype-dependent exudation patterns can strongly shape rhizosphere microbiome assembly, emphasizing reciprocal interactions between plant physiology and microbial recruitment (Garrido-Sanz and Keel, 2025). In particular, the enrichment of *Pseudomonas* may be an adaptive response to Fe limitation, as this genus is known for siderophore-mediated Fe acquisition (Zhao et al., 2024). Ryegrass, conversely, promoted a more stable community dominated by genera like *Lbysoacter* and *Vicinamibacteraceae* across stages, implying a consistent influence on microbes involved in nutrient solubilization (e.g., P, K). A core finding of this study is the correlation between *Pseudomonas* enrichment, increased soil available Fe, and improved fruit quality (elevated TSS and Vc) under white clover. Among cultured siderophore-producing bacteria, *Pseudomonas* was the dominant genus (79.78%), and one strain, PA9, was strongly linked to improved Fe uptake and fruit quality. Its inoculation enhanced root Fe acquisition, prevented a decline in leaf Fe during fruit maturation, and significantly increased chlorophyll content, fruit TSS, and Vc. This supports a model in which white clover fosters siderophore-producing *Pseudomonas* strains that enhance Fe mobilization, thereby supporting photosynthetic performance and carbon allocation to fruits. Ryegrass, while also improving fruit quality, likely operated through alternative pathways, possibly via improved P/K availability and a different microbial consortium, as its enriched microbes showed stronger correlations with nutrients other than Fe.

It should be noted that white clover (a legume) and ryegrass (a grass) differ fundamentally in their physiological traits. Legumes such as clover fix atmospheric nitrogen via symbiosis with rhizobia and secrete flavonoid-rich root exudates, which can selectively recruit specific microbial taxa, including siderophore-producing Pseudomonas. In contrast, grasses like ryegrass prefer nitrate as a nitrogen source and exhibit distinct carboxylate exudation profiles. A recent study in rice directly demonstrated that host metabolites (e.g., phenylpropanoids) explain a substantial proportion (up to 35.6%) of microbiome variation across different genotypes, supporting the view that plant-specific root exudates are key drivers of microbial community assembly (Su et al., 2025). These differences may contribute to the distinct microbiome assembly patterns observed in the two cover crop treatments. However, the current study was not designed to dissect the causal relationships between plant physiological traits and microbiome recruitment.

It should also be clarified that the greenhouse used in this study is a standard plastic polytunnel, not an environmentally controlled growth chamber. It has no active temperature or humidity control; temperature fluctuates with external conditions, and freeze-thaw cycles still occur during winter. The only difference from open-field systems is the exclusion of direct rainfall by the roof, which reduces leaching. Regular irrigation was applied to maintain soil moisture. Therefore, while reduced leaching may prolong the residence time of root exudates and microbial inoculants (e.g., PA9) in the rhizosphere, other key environmental factors (temperature variation, freeze-thaw) remain comparable to those in open fields. This supports the generalizability of our findings beyond protected cultivation, although direct validation under open-field conditions is still warranted.

This study integrates agronomic practice with microbial mechanism to explain cover cropping benefits in protected citrus cultivation. We demonstrate that white clover and ryegrass are both effective yet functionally distinct: the former excels in Fe biofortification and *Pseudomonas*-mediated mobilization, while the latter supports multi-nutrient activation. The identification of *Pseudomonas* PA9 as a key agent linking microbial Fe mobilization to fruit quality provides a microbial resource for sustainable citrus cultivation. Future studies should validate these interactions in field conditions and explore the molecular dialogue between cover crops and microbes to optimize selection and management strategies.

## 5 Conclusion

This study found that cover cropping (particularly with white clover) significantly enriches iron metabolism-related functional microbial communities in the citrus rhizosphere, enhancing soil available iron content and root iron uptake. Furthermore, we revealed that PA9 increases leaf iron nutrition and relative chlorophyll content, ultimately improving the TSS and Vc content of citrus fruits. This research provides a new theoretical basis and practical experience for sustainable citrus cultivation, laying a foundation for further studies on the mechanism by which iron-functional microbes regulate fruit quality under cover cropping systems. It is of great significance for improving fruit quality and efficiency and promoting the sustainable development of the fruit industry.

## Data Availability

The datasets presented in this study can be found in online repositories. The names of the repository/repositories and accession number(s) can be found below: https://ngdc.cncb.ac.cn/gsa, CRA039723.
