# Supplementary material for: Cover cropping enhances fruit quality in protected citrus cultivation by modulating rhizosphere microbiome and iron availability
Source: Front Plant Sci. 2026 Jun 18;17:1836783. doi: 10.3389/fpls.2026.1836783 (PMC13323626; doi:10.3389/fpls.2026.1836783)
Supplement: Supplementary file 1 [file DataSheet1.pdf]

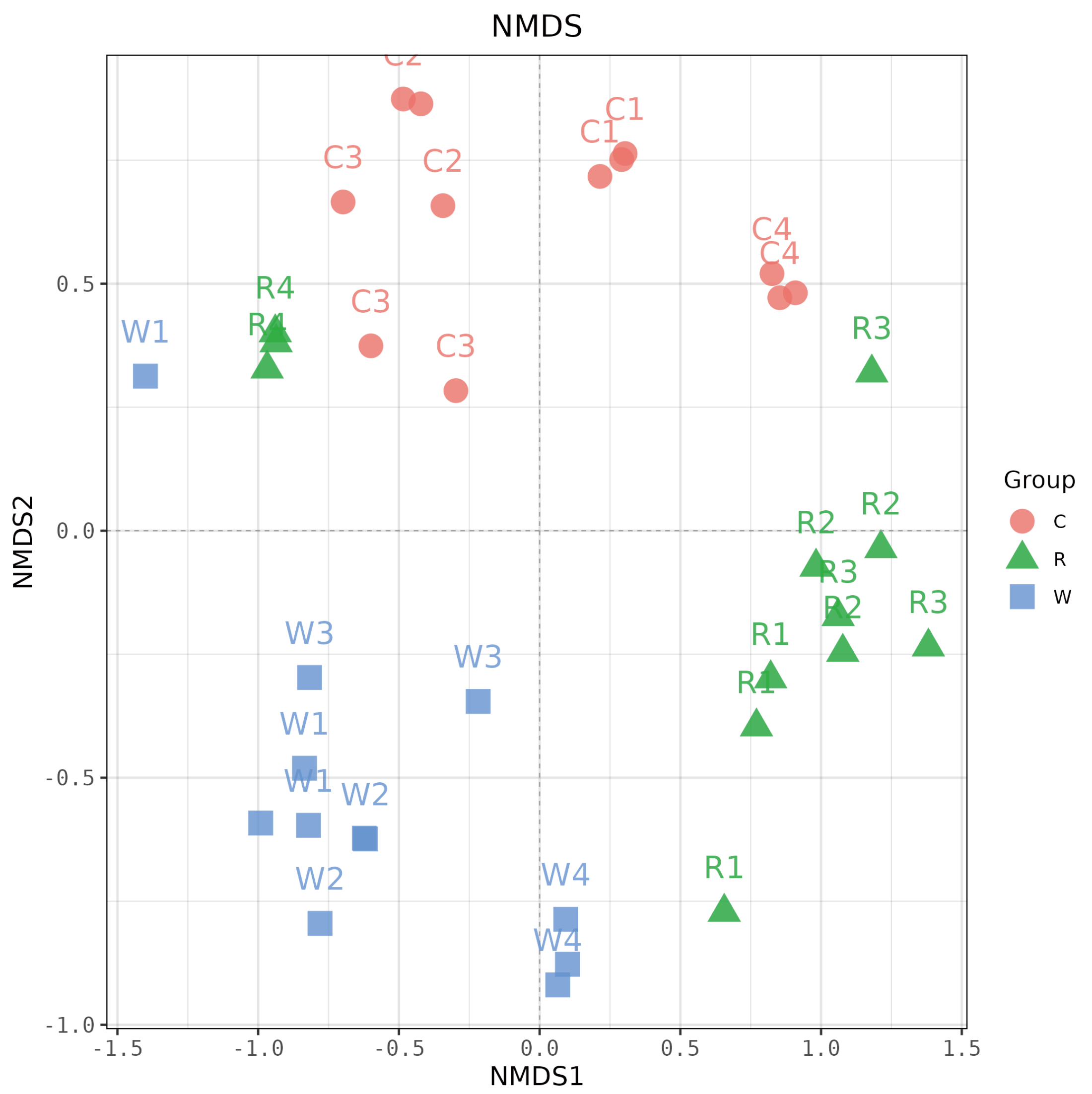

Figure S1. Differences of microbial communities in Citrus rhizosphere soil between cover cropping and clean tillage in polytunnels(the numbers 1, 2, 3, and 4 represent distinct periods, while notations such as W1, R1, and C1 indicate different treatments within the same period).

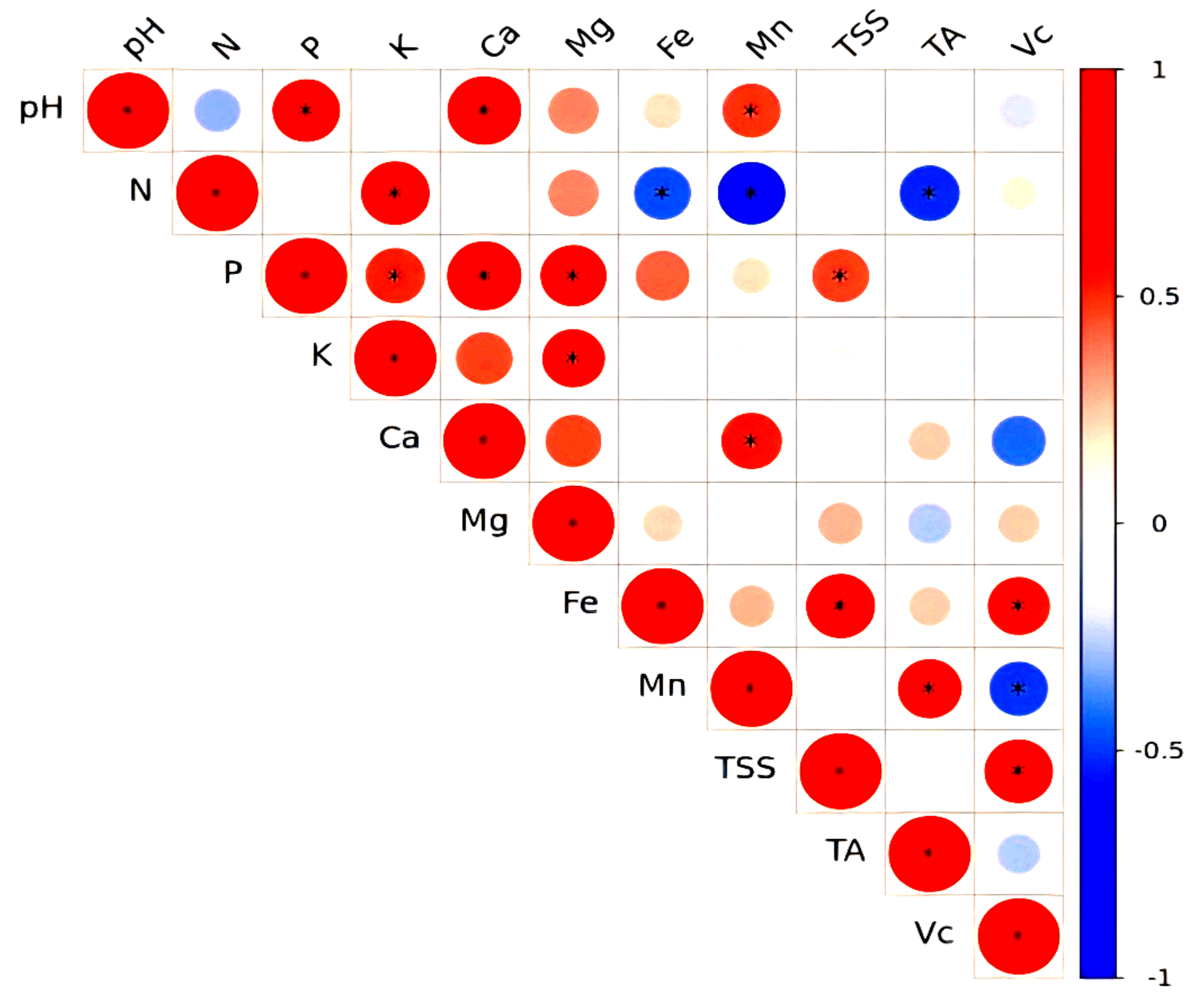

Figure S2. Spearman's correlation analysis between citrus fruit quality and soil physicochemical properties.

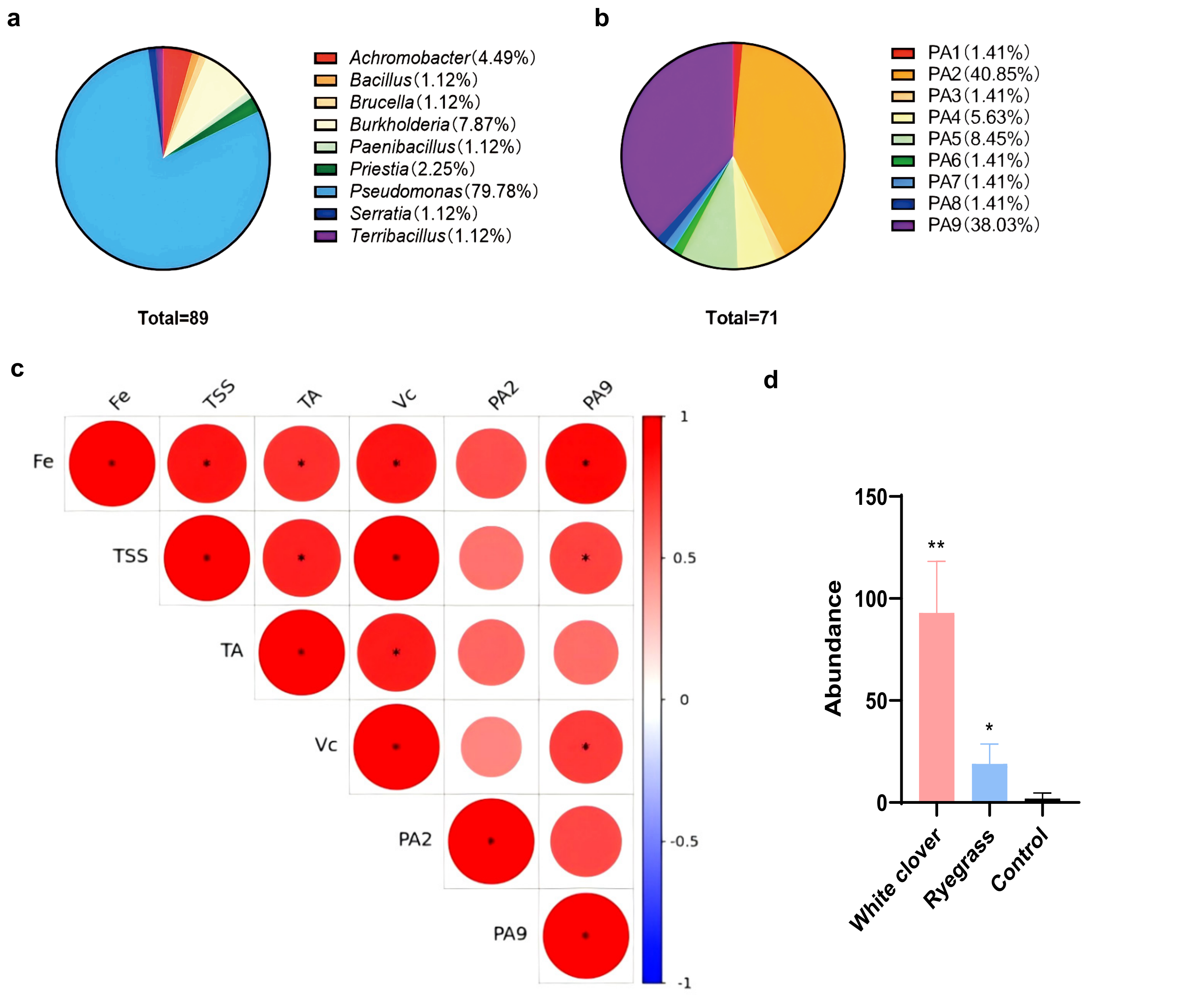

Fig S3. Analysis of rhizosphere microbial composition. **(a)** Relative abundance of bacterial genera successfully detected by gene sequencing. **(b)** Relative abundance of different cultivable Pseudomonas species within the genus. **(c)** Spearman correlation analysis between strains PA2, PA9 and available iron in rhizosphere soil of citrus-producing areas as well as fruit quality. **(d)** The abundance of strain PA9 in citrus rhizosphere soil under different treatments during the fruit ripening stage.
